# Insights Into Ecology, Evolution and Global Change Responses From Very Long‐Term Studies

**DOI:** 10.1111/ele.70072

**Published:** 2025-04-02

**Authors:** Vincent A. Viblanc, Helene C. Muller‐Landau

**Affiliations:** ^1^ CNRS Écologie et Environnement Paris France; ^2^ Université de Strasbourg, CNRS Strasbourg France; ^3^ Smithsonian Tropical Research Institute Panamá Republic of Panama

## 1 The Importance of Long‐Term Studies in Ecology and Evolution

At a time where human activities are altering ecosystems and their associated biodiversity at unprecedented scales (Díaz et al. 2019; McFadden et al. 2023), there is a pressing need to understand how ecosystems function and how they have changed over time, to pinpoint the key processes structuring populations, communities and ecosystems, and to predict how ecosystems and the species inhabiting them will respond to ongoing change. Very long–term studies are crucial because many ecological and evolutionary processes unfold over long time scales, and because the links between cause and effect may take years to manifest (Table 1).

**TABLE 1:** Typical time scales of selected ecological and evolutionary processes compared with those of ecological and evolutionary research.

Typical generation times of organisms	
Microbes	Hours to weeks
Arthropods	Weeks to months
Vertebrates	Years to decades
Herbaceous plants	Months to years
Woody plants	Decades to centuries
Periodicity of major climate drivers	
El Niño Southern Oscillation (ENSO)	2–7‐year periodicity
Pacific Decadal Oscillation (PDO)	20–30‐year periodicity
Atlantic Multidecadal Oscillation (AMO)	60–80‐year periodicity
Timescales relevant for measuring ecological and evolutionary processes	
Duration of seed dormancy	Months to decades
Dormancy of microbial spores	Months to centuries
Duration of secondary succession in perennial plant communities	20–300 years
Lifespans of longest‐lived members of a population	2–50 times the average generation time
Evolutionary responses to change in selective pressure	10–50 generations or more
Typical timespans of data collection	
Undergraduate thesis	Weeks to months
Master's thesis	3–12 months
PhD dissertation	1–3 years
Typical major grant	1–2 years
Long‐term research grant	5–10 years
Very long‐term study	20–50 years
Other research‐relevant timescales	
Length of a scientific career	30–60 years
Funding program lifespan	5–40 years
Age of major funding agency	20–80 years
Age of major field station	50–150 years
Age of major university	50–1000 years

**FIGURE 1:**
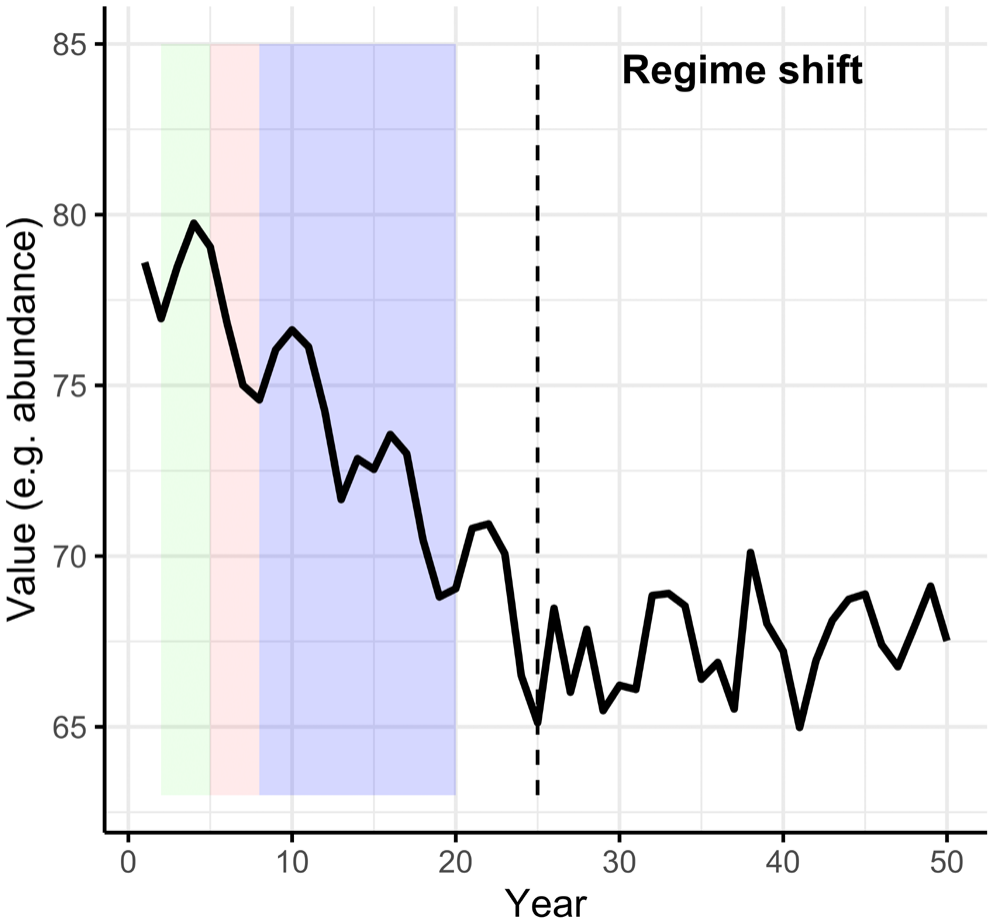
To illustrate how long‐term studies may uncover ecological phenomena that short‐term series cannot, consider a 50‐year time series with a sinusoidal pattern and random noise. Such a time series may, for example, represent the cyclic patterns in population abundance of some species. Because of the cyclic nature of the temporal variation, short‐term studies would come to differing conclusions on temporal trends, depending on when they were conducted: a 3‐year study early on in this example (green box) would find an increasing trend, whereas a 3‐year study started just a few years later (red box) would find a decreasing trend. Both short‐term studies miss the bigger cyclic pattern that could have been captured with a 12‐year study (blue box), itself also showing a long‐term decrease. At 25 years, a regime shift occurs, after which the time series ceases to decrease and its cyclic pattern changes as the variance in the data increases. This regime shift could be detected in very long‐term data spanning the 25‐year threshold but would be entirely missed by shorter time series.

**FIGURE 2:**
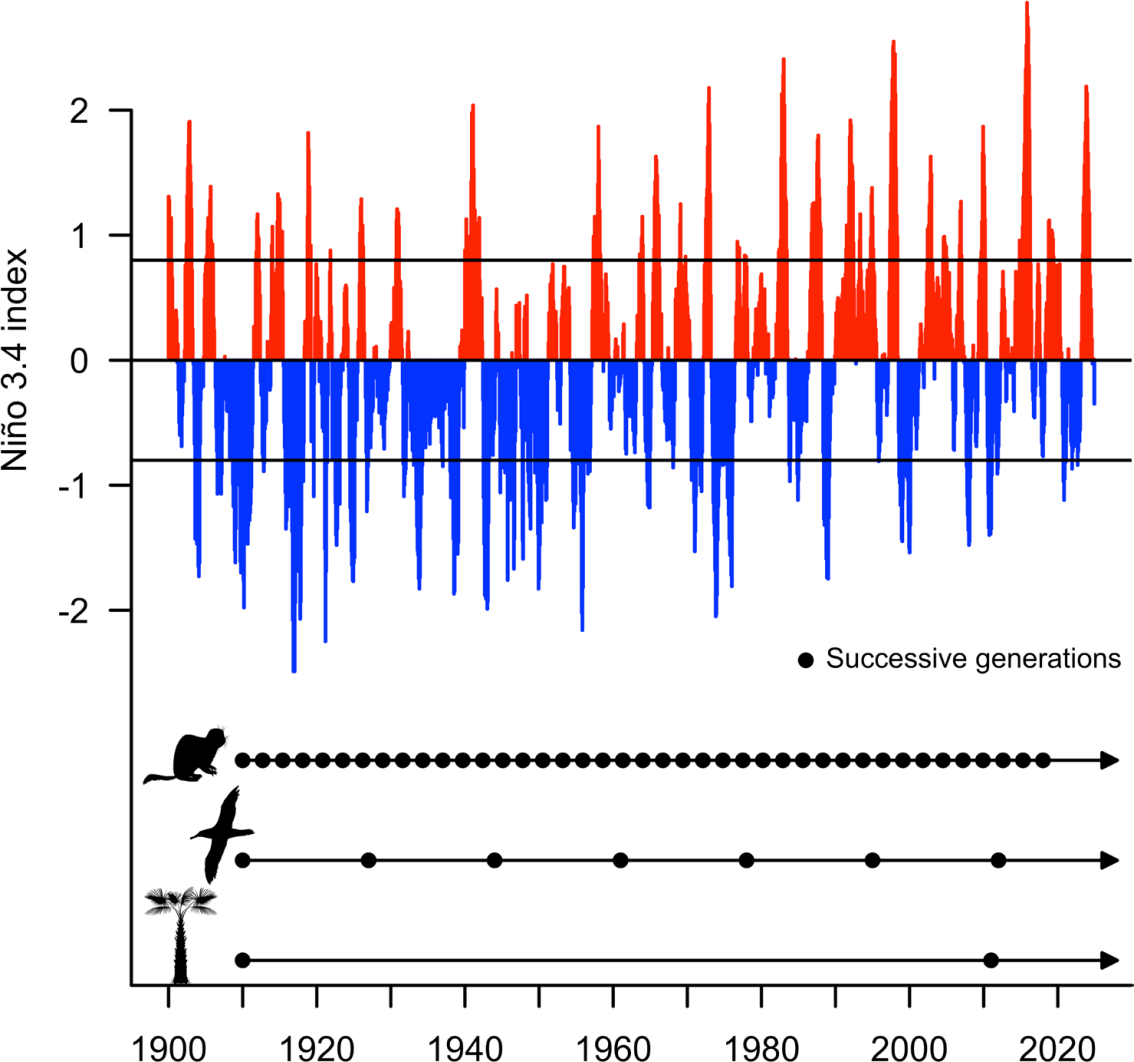
Long‐term cyclical climate variation differently affects species with different generation times, as is illustrated here by considering how temporal variation in the El Niño/Southern Oscillation (ENSO) phenomenon aligns with the generation times of three species. ENSO affects climate globally, and displays an irregular cycle of relatively warm (El Niño, above +0.8°C of the long‐term mean) and cool (La Niña, below −0.8°C) phases with a period of 2–7 years. Consider the order of magnitude difference in generation time between the tropical palm ( *Euterpe globosa* ; generation time estimated at 101 years; Petit and Hampe 2006), the wandering albatross ( *Diomedea exulans* ; 17 years; Milot, Weimerskirch, and Bernatchez 2008) and the golden‐mantled ground squirrel ( *Callospermophilus lateralis* ; 2.7 years; Hostetler et al. 2012). In relatively short‐lived species such as the ground squirrel, a single generation experiences only a small subset of the temporal variation, whereas in long‐lived species such as the palm, a single individual may experience the full breadth. In long‐lived species, the full consequences of early life conditions for individual fitness and population outcomes may take decades to manifest. In short‐lived species, the same scale of temporal environmental variation may lead to greater fluctuations in population abundances, and associated fluctuating selective pressures may also play out in stronger temporal variation in gene frequencies. In both cases, long‐term studies are crucial to understanding population responses to environmental conditions and in unravelling the evolutionary mechanisms (microevolution, plasticity) shaping adaptive responses to environmental change. Data for the Niño 3.4 index (central equatorial Pacific): 5° N–5° S, 120°–170° W was obtained online from the Australian Government Bureau of Meteorology and can be downloaded at http://www.bom.gov.au/climate/enso/index.shtml?ninoIndex=nino3.4&index=nino34&period=monthly#overview‐section=Monitoring‐graphs. Icons CC BY 4.0 downloaded from Creazilla.com (author: Natasha Sinegina, Creazilla).

Long‐term studies enable not only documentation of changes that may be missed (or misperceived) in shorter term snapshots (Figure 1) but also a better understanding of the importance of year‐to‐year variability, including rare events, in shaping ecosystem dynamics and evolutionary responses to change (Franklin, Bledsoe, and Callahan 1990). Recent meta‐analyses demonstrate the importance of long‐term studies to correctly detect the direction of population trends (White 2019; Wright and Calderon 2025) and effects of experimental treatments (Cusser et al. 2021). Further, long‐term studies allow for a closer integration between the fields of ecology and evolution (Jarne and Pinay 2023) as longer datasets increasingly capture evolution in action (e.g., Ålund et al. 2024; Johansson et al. 2024), thus enabling investigations of the roles of fundamental evolutionary processes such as phenotypic plasticity and maternal effects in adapting to change (Carroll et al. 2007; Räsänen and Kruuk 2007; Clutton‐Brock and Sheldon 2010; Charmantier, Garant, and Kruuk 2014). Critically, such evolutionary responses are likely to differ substantially depending on organisms' pace of life and the environmental conditions encountered over individual lifetimes (Figure 2). Of course, even the longest observational and experimental studies are limited by the time scales of the human research endeavour, which are shorter than those of many ecological and evolutionary processes (Table 1).

By collating a series of papers on very long‐term studies in ecology and evolution, this special issue of *Ecology Letters* highlights the contributions of such studies to advancing our understanding of ecological processes and evolutionary mechanisms. Here, we summarise key findings from the papers in this special issue, grouping them into six areas of research: (1) ecological responses to temporal climate variation and climate change; (2) tipping points and regime shifts; (3) synchrony, stability and stationarity; (4) demographic and environmental determinants of population growth rates; (5) evolutionary patterns and potential and (6) causes and life‐long consequences of behaviour. We close with a discussion of their potential to provide structure for inclusive education and professional development, a topic addressed at a greater length by Czapanskiy et al. (2024).

## 2 Contributions of Long‐Term Studies in this Special Issue

### 2.1 Ecological Responses to Temporal Climate Variation and Climate Change

A key strength of very long‐term studies is their ability to document how species, communities and ecosystems respond to temporal climate variation, including long‐term directional climate change. Interannual climate variation is affected by multiple global climate oscillations acting on different timescales, including the El Niño Southern Oscillation (2–7 years), the Pacific Decadal Oscillation (20–30 years) and the Atlantic Multidecadal Oscillation (60–90 years) (McCabe and Palecki 2006). These oscillations, together with the directional forcing due to increasing atmospheric carbon dioxide, mean that climate variation is temporally autocorrelated at a range of timescales and that studies of just a few years invariably fail to capture the breadth of climate variation (Figure 2). Because ecology is highly sensitive to climate, ecological conclusions based on short‐term studies are inherently fragile.

Climate‐related changes in phenology—the seasonal timing of life cycles—have been particularly well studied (Parmesan 2006; Chmura et al. 2018; Inouye 2022). Insofar as such phenological shifts are asynchronous among interacting species, they may alter competitive and trophic dynamics, affecting population performance (Iler et al. 2021) and overall ecosystem function (Beard et al. 2019). In this special issue, Fournier et al. (2024) analyse the covariance of > 2000 long‐term time series of monthly data on the seasonal timing of peak abundance of fish, zooplankton and phytoplankton, together with changes in temperature and salinity, across three major estuarine ecosystems in North America over a period of ca. 30 years. They found that over a quarter of the studied taxa showed significant shifts in phenology, the vast majority advancing their phenology over time in response to changing temperature and salinity. However, 29%–68% of taxa within each trophic level did not appear to be tracking environmental changes in temperature or salinity through measurable phenological responses. In addition, phenological trends sometimes diverged substantially within and among trophic groups (e.g., fish and zooplankton communities) within regions, indicating high potential for trophic mismatches that could disrupt food‐web dynamics.

Besides trophic mismatches, climate‐induced phenological shifts may cause developmental mismatches in some taxa, possibly leading to the so‐called ‘ecological traps’ (Schlaepfer, Runge, and Sherman 2002). For ectothermic insects that rely on temperature to complete critical stages of their life cycles, extended thermal windows for larval development may enable an increase in the number of generations per season (i.e., voltinism, Altermatt 2010). However, because cues from a warming environment may conflict with actual seasonal change as defined by photoperiod, insects run the risk of losing a generation if the season ends before development of the final generation is completed, the larvae then not having undergone the necessary physiological preparations for diapause or winter dormancy (the ‘lost generation’ hypothesis; Van Dyck et al. 2015). Here, Wepprich, Henry, and Haddad (2025) test for the potential of ‘lost generations’ across a long‐term study spanning 27 years and 30 multivoltine butterfly species. Voltinism shifts were overall beneficial to the majority of butterfly species: shifts towards larger last generations generally positively affected species' overwinter population growth rates and long‐term population trends. ‘Lost generations’ were rare, occurring only under limited environmental conditions in a few species. Nonetheless, abundances declined over time in the majority of species, emphasising the need for continuous long‐term monitoring and further investigations into the underlying causes of decline.

Very long‐term studies are also critical to capturing the effects of extreme events, and quantifying the magnitude and duration of their effects. Using long‐term time series spanning over 30 years of fisheries investigations and a US. long‐term ecological research (LTER) site, Chen et al. (2024) study the consequences of multiple marine heat waves of varying intensity and duration for phytoplankton, zooplankton and fish in the southern California current ecosystem. Half of the 30 groups analysed exhibited statistically significant responses to heatwaves, with 14 of 15 showing negative responses, and variation among groups in whether they responded more strongly to intensity or duration. Further research is needed to elucidate the consequences of these variable responses for trophic interactions.

Not only may species differ in their climate responses depending on their own traits, but these responses may themselves depend on the traits and responses of interacting species. Using 30 years of data on growth of tropical trees of 89 species in 15 Amazonian plots, Nemetschek et al. (2025) showed that neighbourhood functional composition and diversity affected tree growth and its response to climate anomalies. Greater functional diversity (trait dissimilarity) among neighbours promoted tree growth. Further, neighbourhood interactions and functional diversity mediated community‐level effects of above average maximum temperature, vapour pressure deficit and climatic water deficit. At a community level, water‐demanding species increased climatic stress for water‐saving species by decreasing water availability, whereas water‐saving species reduced climatic stress for water‐demanding species.

Effects of climate variation may be mediated through multiple climate variables and pathways, as found by Vleminckx et al. (2025) in their 22‐year study of seed production in an ever‐wet Amazonian forest. Despite moderately positive effects of higher daytime temperatures, community‐level seed production decreased due to direct and indirect (via flower production) effects of increasing nighttime temperature and decreasing water vapour pressure deficit. Rainfall also affected seed production, with contrasting effects depending on seasonal timing: rainfall early in the fruiting year reduced same‐year seed production, whereas rainfall at the end of the fruiting year increased the next year's seed production. These effects are important not only for plant regeneration but also for the many species of pollinators, frugivores and granivores that rely on flowers, fruits and seeds for food.

Improved understanding of how climate variation influences demographic performance and abundance at population and community levels is important not only for fundamental ecological understanding but also because of its relevance for conservation and management. For example, very long‐term studies documenting such climate responses can be used to parameterize models to project responses to future climate change scenarios. This is what Jeglinski et al. (2024) did in a study drawing on 116 years of abundance data (1900–2016) for the Northeast Atlantic metapopulation of the northern gannet and fecundity data for a subset of those years.

### 2.2 Tipping Points and Regime Shifts

Given the ubiquity and magnitude of anthropogenic pressures, it is critical to understand the resilience or vulnerability of natural ecosystems to disturbance and change (Holling 1973). What are the tipping points (if any) beyond which regime shifts occur and systems transition to alternative equilibria with different functional and structural organisations? Studies that encompass multiple disturbances and their aftermaths are uniquely well positioned to shed light on these questions. Graham et al. (2024) investigated resilience of coral reefs across the inner Seychelles islands following two marine heatwaves and associated mass coral bleaching events occurring some 20 years apart, through analyses of data on hard corals, soft corals, macroalgae and fish. The first heat wave and mass mortality event were followed by recovery in some coral reefs and a regime shift to macroalgal domination in others. The reefs that recovered from the first mass mortality event were more resistant to community change and recovered more rapidly from the second mass mortality event than those having undergone regime shifts, suggesting an increase in ecosystem resiliency following repeated stressors through time. Graham et al. (2024) also reported a regime‐shift reversal, one of the reefs transitioning back to a coral‐dominated regime following the second heatwave, further evidence that some coral ecosystems may be more resilient than previously thought.

Riginos et al. (2024) address resilience in a savanna ecosystem in Kenya, drawing on 29 years of results from a long‐term large herbivore exclosure experiment to synthesise lessons learned on short‐ and long‐term ecosystem responses to multiple stressors, including drought, herbivory pressure and wildfire. The experimental treatments restricted access of one, two or three groups of large herbivores: cattle, wild mesoherbivores (25–800 kg) and wild megaherbivores (> 1000 kg). The different exclosure treatments resulted in different significant shifts in herbaceous communities and in soil chemistry, which were only detectable after 5–10 years and after 20 years, respectively. The long‐term nature of the study was critical to capture oscillating patterns in ecosystem dynamics contingent on rainfall, in particular related to the competition–facilitation balances between cattle and wildlife, and between different plant species. Temporal climate variation interacted strongly with experimental treatment: the first rainy year after each drought proved an important inflection point for accelerating changes in plant community composition contingent upon the herbivore assemblage. The accumulation of data has recently enabled analyses of longer term ecosystem stability and resilience.

Together, these studies show how long‐term monitoring of natural ecosystems and long‐term experimental treatments importantly complement each other in identifying tipping points in ecosystem function.

### 2.3 Synchrony, Stability and Stationarity

Ecological populations, communities and ecosystems vary over time due not only to temporal variation in climate and other exogenous factors but also in many cases to endogenous dynamics (e.g., predator–prey cycles). The nature and degree of temporal variation, its causes and its consequences are of fundamental interest to ecologists, and also important for understanding the resilience and vulnerability of species, communities and ecosystem services, and thus for conservation and management. Key questions include interrelationships among temporal trajectories of interacting species, whether or to what degree temporal variation is synchronous in co‐occurring species within sites or over sites within species, and how variation in synchrony with diversity and environmental factors translates to temporal variation (or stability) in sums across species or sites and thus to diversity–stability relationships (Loreau and de Mazancourt 2013; Thibaut and Connolly 2013). If multiple species vary in synchrony, then community and ecosystem temporal variation will be higher than if species vary asynchronously. The more species differ in their responses to climate and other factors that vary over time, the more we expect asynchrony. More diverse communities are hypothesised to include more variation in responses, contributing to a ‘portfolio effect’ that reduces variability and increases stability in whole‐community metrics, contributing to positive diversity–stability effects (Tilman, Isbell, and Cowles 2014; Winfree 2020).

Although many studies of synchrony focus on abundance, LaMontagne et al. (2024) evaluated the synchrony of seed production among 103 woody plant species within seven LTER sites. Temporal synchrony varied widely from asynchronous to highly synchronous. Synchrony was greater for species sharing similar traits, but not those more phylogenetically related. Synchrony was also higher in environments characterised by higher water deficits, perhaps because drought conditions made reproduction more difficult or less rewarding in most or all species, concentrating community seed production in non‐drought years. This study builds on many prior single‐taxa studies documenting mast seeding, defined by high and synchronous (within species) temporal variation in seed production. Investigations into the synchrony of seed production in multiple co‐occurring species are rare and shed new light on hypothesised drivers, as well as providing the larger context needed to understand consequences for stand‐level plant regeneration and food availability to consumer species.

Three studies in this special issue investigated synchrony in abundance fluctuations, stability of whole‐community totals and diversity–stability relationships. All three exemplified the difficulty of elucidating the causal relationships of diversity to stability from studies in which diversity is not controlled but instead is an emergent property that depends on other factors that also influence stability. Tsai and Connolly (2025) investigated synchrony and stability in fish communities on the Great Barrier Reef, drawing on data from a 27‐year time period for 39 communities, integrated in a rigorous modelling approach informed by the biology of the system. The authors decomposed the variability of whole‐community abundance into two terms: synchrony and average population variability (following Thibaut and Connolly 2013). They found that both contribute substantially to explaining among‐community variation, with synchrony contributing more. Consistent with hypothesised benefits of diversity for stability, community‐level variability and synchrony were lower in communities with higher richness, but this association was ultimately driven by environmental influences. In particular, reefs closer to the coast and thus more exposed to anthropogenic nutrient inputs and other disruption had decreased richness, increased synchrony and increased community‐level fluctuations, whereas more pristine reefs farther from the coast had higher richness, lower synchrony and greater stability. This study adds to the body of evidence that anthropogenic impacts often affect many species similarly, thereby increasing synchrony and reducing community‐level stability.

Rodrigues et al. (2025) also addressed relationships among species richness, synchrony, population stability and community stability using very long‐term observational data for multiple communities. They analysed 21–40 years of data from many sites in Finland for six groups: birds, butterflies, moths, phytoplankton, small mammals and large mammals. Patterns and inferred relationships varied substantially among taxonomic groups. For example, asynchrony explained more among‐site variation in community stability than did population stability in birds, butterflies, moths and phytoplankton, whereas population stability contributed more in small and large mammals.

Experimental studies offer another avenue for investigating environmental influences on diversity, stability and synchrony. Davidson et al. (2025) took advantage of a long‐term experimental study in a grassland to examine how plant diversity, synchrony and stability are jointly shaped by nutrient addition—a global change driver. Effects differed between an initial ‘transient’ period of 7 years and a later ‘post‐transient’ period spanning 12–22 years after experimental initiation. The final structural equation models for the post‐transient period include significant negative effects of nitrogen addition level on richness, evenness and stability, and of synchrony on stability, but no links from richness or evenness to synchrony or stability; results for the transient period differed. Thus, the results highlight the importance of long‐term studies and also show how the expected covariation in diversity, synchrony and stability can emerge from independent effects of an environmental variable, and need not indicate support for hypothesised effects of diversity on stability.

Whereas the above studies analyse spatio‐temporal data on species abundances to evaluate synchrony within a particular guild, Yazdanian et al. (2024) combined such data for two guilds to investigate trophic dynamics. They drew on bird and moth abundance data for Finland (datasets also used by Rodrigues et al. 2025), combined with functional trait data, to calculate total biomass by functional group and by site, and test hypotheses regarding the relationship of insectivorous bird abundance to moth abundance. Of the 99 resulting tests for influences of moth group biomass on subsequent‐year bird group biomass in a given region, only five were significant at the chosen *p* = 0.002 individual‐test significance level, of which two were unexpectedly negative. Their results suggest that temporal variation in bird abundance is not consistently related to moth abundance as might be predicted from bottom‐up effects alone.

In contrast to the above studies quantifying synchrony (or the lack thereof) among species within sites, Lindenmayer et al. (2024) focused on synchrony across sites within species. They analysed species site occupancy for 18 Australian bird species, specifically testing for differences in temporal trajectories among four long‐term datasets, each encompassing over 100 sites, and together spanning some 1000 km of latitude across eastern Australia. Using Bayesian occupancy modelling accounting for differences in detection probability associated with weather conditions and observer ID, they found significant among‐study variation in temporal patterns for 14 of the 18 species, indicating a lack of synchrony or non‐stationarity. Contrasting with the spatio‐temporal pattern of synchrony, non‐stationarity occurs when the effects of a given factor (e.g., climate) on an ecological process (e.g., population abundance) vary across space and/or time. Non‐stationarity in occupancy was not related to the distance from a species' niche centroid, nor was it related to bioclimatic variables (temperature and precipitation). These results highlight that responses of species trajectories at global scales may differ substantially from those observed at local spatial scales, with important implications for management practices.

### 2.4 Demographic and Environmental Determinants of Population Growth Rates

One fundamental goal of ecology is understanding the factors shaping population growth rates, notably the demographic factors causing populations to grow and decline. Analyses of population growth rates typically assume that a population's age (or stage) distribution is stable through time and thus that population growth can be estimated through its long‐term asymptotic measure of growth rate (Charlesworth 1994; Caswell 2001). However, most ecological environments are variable by nature, and stochastic events (e.g., storms, wildfires) may lead to marked changes in a population's demography. In such cases, the asymptotic growth rate may differ substantially from the short‐term population growth rates. The development of transient life table response experiments (tLTRE) addresses this challenge by quantifying the contribution of population vital rates to population growth rates, while accounting for temporal variation in vital rates and demographic structure (Koons et al. 2016). In this issue, two papers use tLTRE to mechanistically link environmental and demographic variables to realised population growth rates.

Dobson et al. (2024) quantified the role of demographic versus environmental stochasticity on population growth rates during periods of population increase and decline, and tested the hypothesis that traits with the greatest influence on population growth should exhibit the least amount of variation over time (‘demographic buffering’, Gaillard and Yoccoz 2003; Hilde et al. 2020). In a Columbian ground squirrel ( *Urocitellus columbianus* ) population that went through an initial rapid 10‐year period of growth, followed by a 2‐year crash and a subsequent 19‐year recovery, they showed that the demographic mechanisms behind rapid population growth or crash were essentially the same and mainly attributable to environmental stochasticity, explaining three‐quarters of the variance in the population growth rate. Contrary to the demographic buffering hypothesis, the primary demographic cause of fluctuations in abundance of was survival, which varied substantially among years.

As habitat fragmentation increases and species become more isolated, immigration is expected to decrease and inbreeding increase, reducing genetic diversity and threatening population long‐term viability (Schlaepfer et al. 2018). Summers et al. (2024) studied the consequences of anthropogenic habitat fragmentation and increasing isolation on the vital rates of vulnerable Florida scrub jay ( *Aphelocoma coerulescens* ) over a 34‐year period. They found that population stability was maintained despite increased isolation and inbreeding owing to compensatory adjustments in vital rates together with density‐dependent immigration. Their results provide rare insights into persistence of a threatened population.

### 2.5 Evolutionary Patterns and Potential

Long‐term studies provide unique opportunities to study eco‐evolutionary dynamics (Pelletier, Garant, and Hendry 2009) and quantify contemporary evolutionary change, including that due to anthropogenic influences. For example, warming climates are hypothesised to select for smaller body size and longer appendages in animals to increase the relative surface area for heat loss (Sheridan and Bickford 2011; Ryding et al. 2021). Accumulating evidence indeed suggests that changes in morphology are occurring in some wild populations, but large‐scale, phylogenetically controlled studies are needed to assess the generality of this phenomenon across taxa (Gardner et al. 2011). McQueen et al. (2024) reported on a 46‐year study of morphological change in 25 Australian shore bird species. Size declines were widespread, with negative trends for body mass in 22 of 25 species and for wing length in 18 of 25 species. In contrast, trends in bill length were highly variable among species, with a tendency towards an increase in relative bill length. Overall, changes in body size were consistent with adaptations to dissipate body heat in warming climates, although phenotypic shifts could have been due to other pressures, such as increased nutritional stress.

Although classically investigation of phenotypic trait inheritance has focused on genetic processes, non‐genetically inherited changes in environments—ecological inheritance—can shape evolutionary responses (Danchin et al. 2011; Danchin 2013). For example, phenotypes able to acquire and monopolise fitness‐enhancing resources (‘material wealth’, e.g., territories with abundant food resources) should have a selective advantage. Wealth inheritance might also occur through direct inheritance (e.g., territory inheritance) or non‐genetic inheritance (e.g., social learning of phenotypic abilities). Understanding how the mechanisms of inheritance operate is important because alleles associated with high material wealth might increase in frequency in populations simply because of the fitness benefits of material wealth without any causal association between alleles and wealth acquisition. Yet, attempts to evaluate the contribution of genetic and non‐genetic patterns of wealth inheritance are rare because long‐term pedigree, genetic and behavioural data are required to statistically partition genetic versus non‐genetic sources of variation (Charmantier, Garant, and Kruuk 2014). Ålund et al. (2024) used a very long‐term study of collared flycatchers ( *Ficedula albicollis* ) to critically evidence non‐genetic inheritance patterns of fitness‐associated material wealth, decoupled from genetically inherited traits. Combining genome‐wide‐association analyses, cross‐fostering experiments, and gene set enrichment analyses and simulations, they found modest (h^2^ ~ 0.2) narrow sense heritability but considerably higher (*h* ^2^ ~ 0.4) genomic heritability of material wealth (in this case, caterpillar larvae biomass), indicating its association with genomic markers. Cross‐fostering revealed stronger associations between foster parents and offspring, supporting non‐genetic inheritance. Importantly, natal dispersal strongly predicted parent–offspring resemblance in material wealth, with philopatry largely responsible for wealth inheritance.

Besides microevolutionary processes, phenotypic plasticity—the ability of a single genotype to produce multiple phenotypes (West‐Eberhard 1989)—is a key feature of adaptation (Pigliucci 2001, 2005; Pfennig 2021). Behavioural traits, in particular, constitute frontline responses to environmental challenges, their plasticity varying within and among populations (Dingemanse et al. 2012; Dingemanse and Wolf 2013). The expression of plasticity across behaviours (plasticity syndromes), together with positive fitness outcomes, would suggest a generalised adaptiveness of behavioural plasticity. Alternatively, plasticity in one behavioural trait may constrain plasticity in another (Sheehy and Laskowski 2023). Johansson et al. (2024) tested these alternatives using a 40‐year data set of repeated behavioural observations on > 1800 Magellanic penguins ( *Spheniscus magellanicus* ) and associated climatic and oceanographic variables. They found positive correlations in plasticities for two reproductive behaviours (egg laying date and mate switch probability) and for two foraging behaviours (foraging trip distance and foraging site fidelity), but a negative correlation between a foraging and breeding behaviour, providing support for both plasticity syndromes and allocation constraints. Relationships between individual behavioural plasticity and fitness varied with environmental conditions, a finding consistent with the maintenance of diverse behavioural plasticities in natural populations.

### 2.6 Causes and Life‐Long Consequences of Behaviour

Individual‐based long‐term studies enable investigation of the proximate causes and ultimate fitness outcomes of particular behaviours. The strength of long‐term studies is that they have the unique power to analyse fitness outcomes of behavioural strategies over the lifetimes of individuals, as well as to illuminate the degree to which frequencies of behaviours vary over time, for example, in response to changing environmental conditions. This is particularly important for understanding the adaptive value of behaviours that occur repeatedly over the lifetimes of individuals such as mate switching. Deliberate mate switching or ‘divorce’ has been observed in the vast majority (> 90%) of monogamous species and occurs at highly variable rates among species (Jeschke and Kokko 2008). Conclusions on its adaptiveness, however, depend on the methodology used to study the fitness consequences of such a strategy (Culina, Radersma, and Sheldon 2015). Measuring fitness the season after partner switching, for instance, may be misleading as mate switching per se (not divorce) may be costly. Drawing on a 24‐year individual‐based monitoring program of Seychelles warblers ( *Acrocephalus sechellensis* ), Speelman et al. (2024) analysed 1063 partnerships, of which 69% ended in widowhood and 14% in divorce, with some individuals divorcing up to three times in their lifetime. Causes for divorce included low reproductive output the preceding season, pair‐bond tenure and male age (higher divorce rates in young and old individuals, possibly attributable to lack of experience and senescence, respectively). Divorcees did not exhibit higher short‐ or long‐term fitness. Divorced females that lost their resident breeding position early in life fared worse in terms of later life survival than females that never divorced, showing that the fitness consequences of divorce may be sex/status dependent. Interestingly, a minority of individuals stepped down from breeder positions upon divorcing to act as helpers for related dominants.

Using a Bayesian approach, Sun et al. (2024) analysed the life history outcomes of divorce and widowhood over a 54‐year long‐term study of snow petrels ( *Pagodroma nivea* ). Despite annual probabilities being generally low (less than 8%), divorce and widowhood carried substantial fitness costs. Whereas divorcees improved subsequent breeding success after mate switching compared to past attempts, mate switching entailed lower breeding probabilities and survival costs both for divorced and widowed birds, impacting long‐term fitness. Critically, widowhood and divorce rates were affected by environmentally harsh conditions both through direct effects on the birds' decision‐making processes and indirect effects mediated through survival or breeding failure. Forecasting future climate trends using the CESM2 Large Ensemble projections, the authors further showed that male widowhood probability is anticipated to increase, possibly resulting in skewed operational sex ratios with likely consequences for population demography.

Another important behaviour in many species of birds and other animals is territoriality, which influences individual fitness and population trajectories. Zammarelli et al. (2024) evaluated how territory sizes change with abundance among years through analyses of 52 years of territory maps for seven migratory songbird species in a 10‐ha plot of temperate forest. In all species, territory sizes declined in years of higher abundance, and did so similarly in both more and less preferred areas, consistent with predictions of the ideal free distribution. The authors thus rejected the ‘ideal despotic distribution’ hypothesis that dominant individuals (‘despots’) are able to maintain large favourable territories even in years of greater conspecific abundance and competition. These findings are relevant for wildlife conservation and management, as conformity with the predictions of the ideal free distribution is expected to contribute to stronger density‐dependent regulation of populations and reduced risk of stochastic extinction.

## 3 The Broader Value of Long‐Term Studies

Long‐term research programs can provide unique structures for inclusive education and professional development, as discussed by Czapanskiy et al. (2024). Drawing upon 60 years of experience on a long‐term study of northern elephant seals ( *Mirounga angustirostris* ), they identified four key points that make long‐term studies catalysts of inclusive education. (1) Rich data sets enable in‐depth analytical and question‐oriented training, often with a level of complexity beyond that typically expected from shorter term undergraduate research projects. (2) Well‐established and well‐equipped research infrastructure provide inclusive entry points into the field of ecology. Both of the above critically allow trainees to build research and self‐efficacy skills, while contributing to a long‐term research program larger than the individual projects. In addition, long‐term studies facilitate professional development through (3) the establishment of networks between individuals at multiple career stages, both locally (within the research program) and globally (through conferences, mentor networks of collaborators), and (4) creating peer cohorts that foster science identity, confidence and self‐efficacy while reducing isolation and interpersonal conflicts.

Czapanskiy et al. (2024) argued, however, that the reliance of many long‐term research programs on unpaid labour is a critical factor acting against diversity, equity and inclusivity in long‐term studies. Their findings from survey responses of 27 researchers who submitted proposals for this *Ecology Letters* special issue found that whereas long‐term studies almost all relied at least in part on undergraduate students for data collection and/or analyses, less than half offered some sort of financial compensation to students (beyond housing and/or transport). Whereas self‐funded, volunteer work is common in ecological and evolutionary research, this approach is inequitable in that it discriminates against people who cannot afford to work unpaid for prolonged periods of time, which disproportionately affects members of many historically underrepresented groups (Fournier and Bond 2015). Czapanskiy et al. (2024) suggested that one way to navigate the difficulties of acquiring the necessary recurrent funding to support long‐term research work (including by undergraduates and interns) is partnering long‐term projects with institutional and/or national diversity, equity and inclusion (DEI) programs, an approach many funding agencies (e.g., NSF, NSERC, ERC) are explicitly encouraging. By investing financially in a culture of inclusion, very long‐term studies may well initiate a virtuous cycle whereby young people involved in the program are more likely to pursue and obtain jobs in science in the future (Bailey et al. 2022), and thus be in a position to themselves contribute further to the maintenance of long‐term data collection.

## 4 Concluding Remarks

Together, the articles presented in this two–part special issue of *Ecology Letters* clearly show the breadth and depth of contributions, both fundamental and applied, made by very long‐term studies. The datasets and code archived together with the articles in these special issues make them even more valuable, enabling others to more easily build on these contributions with further analyses of these data or application of the same analytical methods to other datasets. Indeed, several of these contributions were highly praised by our data editors for their excellent work in presenting code and data in a clear and reproducible way (e.g., Rodrigues et al. 2025). At the same time, issues of fairness of data publication practices to data originators can be particularly fraught for very long‐term studies (De Lima et al. 2022). This problem is exacerbated by common citation practices that result in a lack of attribution and citation to data‐originating papers (Payne et al. 2012).

Very long‐term studies are crucial for quantifying and disentangling effects of climate cycles and global change, understanding ecological and evolutionary processes that unfold over prolonged periods, providing data to inform and evaluate theory and models, and supporting evidence‐based management and policy (Lindenmayer et al. 2012). Further, such studies, like long‐term field sites, generally become foci for a range of complementary investigations and build communities of researchers, thereby synergistically advancing understanding while providing exceptional learning opportunities (Michener et al. 2009; Muller‐Landau and Wright 2024). At a time when the funding culture of scientific research favors short‐term projects of 3–5 years at best, it is critical to recognise the outstanding contributions of very long‐term studies, and to highlight the importance of continued investment in such research.

## Author Contributions

Vincent A. Viblanc and Helene C. Muller‐Landau co‐wrote the paper.

## Conflicts of Interest

Vincent A. Viblanc co‐authored one paper in the associated special issue.

### Peer Review

The peer review history for this article is available at https://www.webofscience.com/api/gateway/wos/peer‐review/10.1111/ele.70072.

## Data Availability

R‐code and data for the figures are accessible on figshare (doi: 10.6084/m9.figshare.28179902).
